# Circumferential strain and strain rates of the descending aorta as novel measures of aortic stiffness and wall mechanics from standard cardiac MRI

**DOI:** 10.1113/EP092585

**Published:** 2025-05-05

**Authors:** Denis J. Wakeham, Sauyeh K. Zamani, Andrew P. Oneglia, Matthew M. Howrey, Samer Majeed, Tiffany L. Brazile, Joshua A. Beckman, James P. MacNamara, Mark J. Haykowsky, Vlad G. Zaha, Benjamin D. Levine, Christopher M. Hearon, Satyam Sarma, Michael D. Nelson

**Affiliations:** ^1^ University of Texas Southwestern Medical Center Dallas Texas USA; ^2^ Institute for Exercise and Environmental Medicine Texas Health Presbyterian Hospital Dallas Texas USA; ^3^ Department of Kinesiology University of Texas at Arlington Arlington Texas USA; ^4^ Faculty of Nursing University of Alberta Edmonton Canada

**Keywords:** ageing, aorta, arterial stiffness, HFpEF, magnetic resonance imaging

## Abstract

During standard cardiovascular magnetic resonance (CMR) the horizontal long‐axis cine image (i.e., 4‐chamber) is captured which includes a cross‐section of the descending aorta. The aortic cross‐section can be used to assess aortic stiffness (distensibility; ∆area/pressure) or circumferential strain (percentage vascular deformation). We examined whether descending aortic strain from traditional CMR is sensitive to age‐ and disease‐related (heart failure with preserved ejection fraction; HFpEF) arteriosclerosis. We recruited 83 participants into three groups: (1) 34 young individuals (age: 22 ± 3 years; body mass index (BMI): 24.3 ± 2.8 kg/m^2^); (2) 19 older individuals (age: 69 ± 5 years; BMI: 26.9 ± 4.7 kg/m^2^) and (3) 26 patients with HFpEF (age: 69 ± 6 years; BMI: 35.8 ± 6.1 kg/m^2^). All participants were studied in the same 3 T scanner (Phillips, Achieva). Descending aortic cross‐sectional area and circumferential strain were measured using cvi^42^ software. Blood pressure was measured via a brachial oscillometric cuff. Data were compared via ANOVA. All data are reported as means ± standard deviation. Compared to the young group (71 ± 5 mmHg), mean arterial pressure was higher in the older (83 ± 9 mmHg, *P *< 0.001) and HFpEF groups (86 ± 10 mmHg, *P *< 0.001). Minimum and maximum aortic areas were greater in the older and HFpEF groups (both, *P *< 0.01). Peak descending aortic strain (young: 11.4% ± 2.2%; older: 4.8% ± 1.6%; HFpEF 3.8% ± 1.6%) and absolute distension were lower (all, *P *< 0.02) in the older and HFpEF groups compared to the young. Peak descending aortic strain and strain rates are sensitive to age and may provide a novel assessment of arterial stiffness for longitudinal studies that utilize or have utilized CMR.

## Abstract (highlights)

- **What is the central question of this study?**
  Assessing aortic stiffness requires prospective planning when done by magnetic resonance imaging (MRI), but is it possible to assess aortic stiffness from standard cardiac MRI?
- **What is the main finding and its importance?**
  Peak strain and systolic and diastolic strain rates of the descending aorta are sensitive to age‐related aortic stiffening, with less variability than conventional measures. The descending aorta captured in the standard four‐chamber view with cardiac MRI can be used to assess aortic stiffness and wall mechanics. These variables represent novel metrics available from every cardiac MRI ever taken.

## 1 INTRODUCTION

The aorta undergoes arteriosclerosis with ageing, which is characterized by increases in aortic stiffness, diameter, length and tortuosity (Chirinos et al., 2019). Arterial stiffening is an important haemodynamic determinant of central and peripheral arterial hypertension (Chirinos et al., 2019), and is prognostic for future cardiovascular events in health and disease (Ben‐Shlomo et al., 2014; Townsend et al., 2015). Indices of aortic stiffness, such as pulse wave velocity, aortic distensibility and strain, are therefore often measured with a variety of imaging techniques, including magnetic resonance imaging (MRI) (Ohyama et al., 2018). While informative, specific aortic imaging requires prospective planning and additional scan time beyond conventional imaging, which can be costly and increase patient burden.

Assessments of arterial mechanics provide insight into the magnitude and rate of arterial deformation (termed strain and strain rate). The study of vessel mechanics provides further insight into vessel stiffness and function above and beyond the conventional indices of local vessel compliance/stiffness. Indeed, when compared with conventional local measures of arterial stiffness (elastic modulus and β stiffness index), ultrasonographic circumferential strain imaging of the common carotid artery in cross‐section has been suggested as a superior measurement of age‐related arterial stiffening (Bjallmark et al., 2010). Importantly, prospectively planned MRI analysis validated intra‐operative transoesophageal echocardiography assessments of descending aortic circumferential strain (Rong et al., 2020), but this approach required specific aortic imaging. To circumvent the requirement for additional scan time, the horizontal long‐axis cine image (i.e., 4‐chamber view) captured during standard cardiovascular magnetic resonance (CMR) includes a cross‐section of the descending aorta, which may provide a unique opportunity to evaluate arterial stiffness.

The purpose of our study was to determine whether peak circumferential strain of the descending aorta obtained from traditional CMR is sensitive to the known age‐related differences and potential disease‐related (heart failure with preserved ejection fraction; HFpEF) differences in aortic stiffness (i.e., arteriosclerosis). Descending aortic distensibility was used as our conventional index of aortic stiffness to determine the presence of age‐ and disease‐related arteriosclerosis. We hypothesized that peak descending aortic strain would be lower in the older and HFpEF groups, with the lowest strain in the HFpEF group.

## 2 METHODS

### 2.1 Ethical approval

All participants provided written informed consent. We present secondary analysis from two prior studies conducted by our group, both of which were approved by the Institutional Review Board at the University of Texas Southwestern Medical Center (IRB no.: Young group, STU‐112 017‐013; Older and HFpEF groups, STU‐2019‐0617). Our study conforms to the *Declaration of Helsinki*, except for registration in a database.

### 2.2 Study participants

We analysed data from 83 participants from three distinct groups: (1) 34 young healthy adults (22 ± 3 years) (Oneglia et al., 2023); (2) 19 older adults (69 ± 5 years), and (3) 26 patients with HFpEF (69 ± 6 years) (Sarma et al., 2023). Young healthy participants were studied if they presented with a body mass index (BMI) between 18.5 and 30 kg/m^2^, were non‐smokers, normotensive (<140/90 mmHg), not taking any cardiovascular‐acting medications and completing <3 days of vigorous exercise per week; pre‐menopausal women were not studied if pregnant or taking oral contraceptives. Similar criteria were used for the older group, other than participants were excluded if their BMI was >40 kg/m^2^. Patients with HFpEF were recruited for an ongoing clinical trial (NCT04068844) and were included if they were aged ≥55 years, with a history of heart failure hospitalizations and presented with elevated resting (≥15 mmHg) and/or peak exercise pulmonary artery wedge pressure (≥25 mmHg) as assessed via right‐heart catheterization in the seated‐upright position. Participants were also excluded if they met specific exclusion criteria for undergoing an MRI.

### 2.3 Study protocol

**FIGURE 1:**
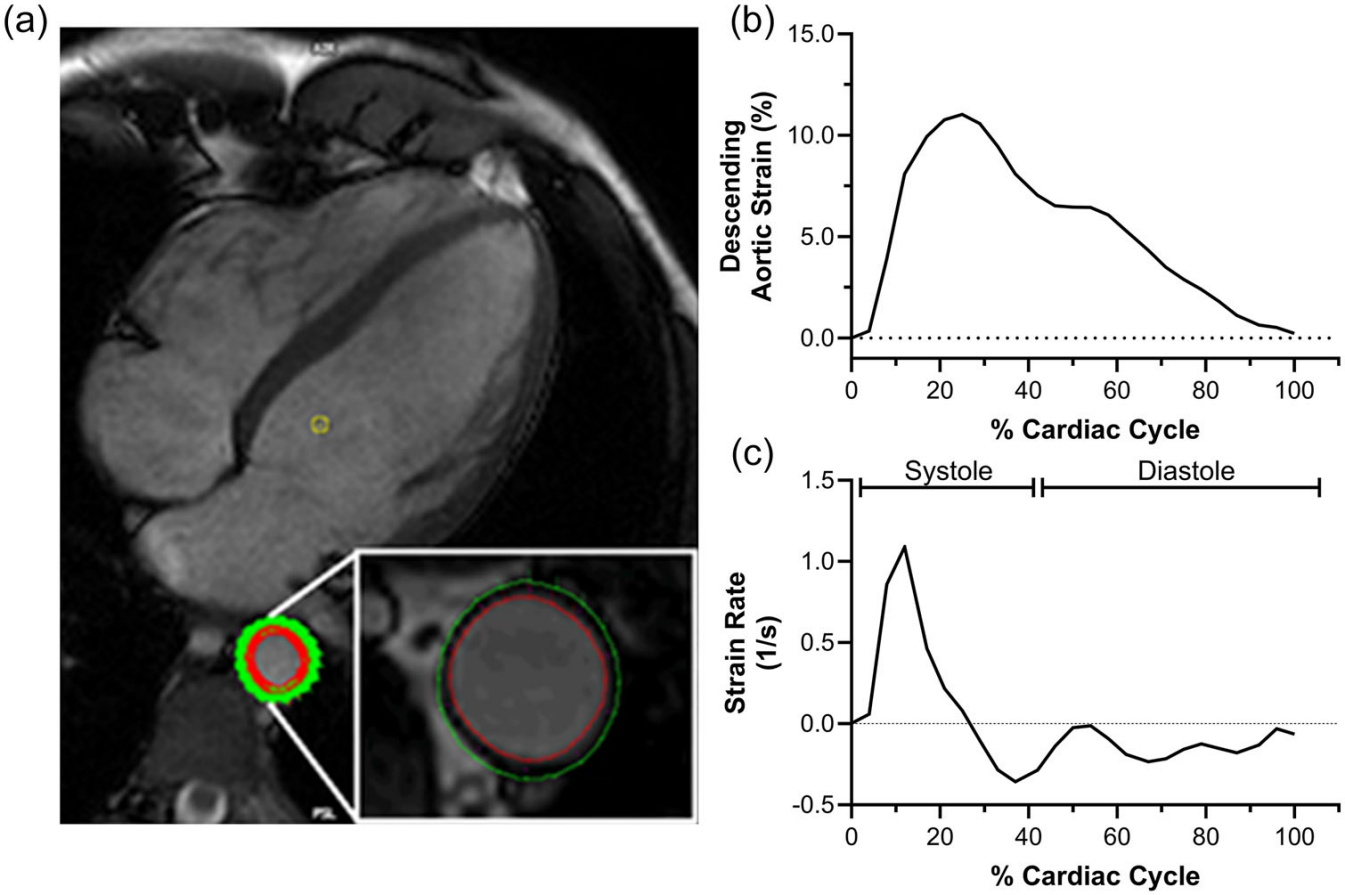
Circumferential strain of the descending aorta. (a) Representative image showing descending aortic strain and strain rate analysis conducted by placing markers around the intimal and adventitial walls to track relative vessel deformation across the cardiac cycle. (b, c) Average descending aortic circumferential strain (b) and strain rates (c) indexed to percentage of cardiac cycle duration.

All participants were studied in the same 3 T magnetic resonance scanner (Achieva, Phillips, The Netherlands). Upon arrival to the Advanced Imaging Research Centre at The University of Texas Southwestern Medical Center, participants were instrumented with an Oscillometric blood pressure cuff around the brachial artery (Philips Invivo Expression Physiology Monitor, Orlando, FL). Following instrumentation, participants laid supine in the scanner during image optimization and localization before study data were captured to assess cardiac morphology and function using the following parameters: 3.0 ms repetition time, 1.49 ms echo time, 40° flip angle, 10 mm slice thickness with a 4 mm gap between slices, 232 × 227 matrix and 350 × 350 field of view. Blood pressure was calculated as the average of at least three blood pressures collected during imaging. Following the study, descending aortic cross‐sectional area and circumferential strain and strain rate were measured from the standard four‐chamber view using Circle Cardiovascular Imaging (Circle CVI) software (V5.13.5; Figure 1). To calculate peak circumferential strain of the aorta, we traced the tunica intima and adventitia manually at end‐diastole and end‐systole, and then tracked the change throughout the cardiac cycle using the strain algorithm within Circle CVI. The software automatically calculates the strain rate, from which we manually extracted the peak/nadir strain rates in early systole, late systole and during diastole. Each strain and strain rate curve was inspected prior to inclusion independently by three investigators (S.K.Z., M.D.N., D.J.W.). Intra‐observer reliability for descending aortic strain was 1.9% (coefficient of variation). We determined maximum and minimum aortic area to calculate descending aortic distensibility as: ([Max Area – Min Area]/Min Area)/(Systolic Pressure − Diastolic Pressure). Stroke volume was determined from the difference between left ventricular end‐diastolic and end‐systolic volume traced manually at end‐systole and end‐diastole for each slice.

### 2.4 Statistical analysis

Following the assessment of normality and conformity of data with parametric assumptions, all data were compared via ANOVA with Tukey's *post hoc* test of multiple comparisons and are reported as means ± standard deviation. The relationship between variables was assessed with Pearson's product‐moment correlation coefficient. The impact of physiologically relevant variables on key outcomes was assessed via ANCOVA. All statistical analyses and graphing were conducted using GraphPad Prism (v10.4.1; GraphPad Software, Boston, MA, USA) other than the ANCOVA (SPSS Statistics, v20; IBM Corp., Armonk, NY, USA).

## 3 RESULTS

The proportion of females was not significantly different between groups (*P *= 0.246), and BMI was greater in HFpEF compared to both other groups (*P *< 0.001; Table 1). Compared to the young group, blood pressures were higher in the older and HFpEF groups (both *P *< 0.001), with no difference between the older and HFpEF groups.

**TABLE 1:** Demographics and MR data.

Variables	Young (*n* = 34)	Older (*n* = 19)	HFpEF (*n* = 26)	ANOVA *P*
Age, years	22 ± 3	69 ± 5^a^	69 ± 7^a^	**<0.001**
Female, %	38	63	57	0.246
BMI, kg/m^2^	24.3 ± 2.8	26.9 ± 4.7^a^	35.3 ± 6.1^a,b^	**<0.001**
HR, bpm	63 ± 8	63 ± 8	64 ± 11	0.891
SBP, mmHg	102 ± 7	119 ± 10^a^	121 ± 12^a^	**<0.001**
DBP, mmHg	56 ± 7	65 ± 10^a^	68 ± 12^a^	**<0.001**
PP, mmHg	46 ± 7	53 ± 11	52 ± 14	**0.038**
MAP, mmHg	71 ± 6	83 ± 9^a^	86 ± 10^a^	**<0.001**
Cardiac				
Stroke volume, mL	83 ± 18	71 ± 18	87 ± 19^b^	**0.019**
Descending aorta				
Min area, cm^2^	2.6 ± 0.7	4.2 ± 0.8	4.7 ± 0.8	**<0.001**
Max area, cm^2^	2.9 ± 0.8	4.4 ± 0.8^a^	4.9 ± 0.8^a^	**<0.001**
Distention, cm^2^	0.36 ± 0.24	0.22 ± 0.07^a^	0.20 ± 0.07^a^	**<0.001**
Distensibility, cm^2^/mmHg	0.0031 ± 0.0021	0.0010 ± 0.0004^a^	0.0008 ± 0.0004^a^	**<0.001**
Peak Strain, %	11.4 ± 2.2	4.8 ± 1.6^a^	3.8 ± 1.6^a^	**<0.001**
Early systolic strain rate, 1/s	1.25 ± 0.30	0.53 ± 0.19^a^	0.41 ± 0.14^a^	**<0.001**
Late systolic strain rate, 1/s	−0.41 ± 0.16	−0.15 ± 0.08^a^	−0.12 ± 0.05^a^	**<0.001**
Peak diastolic strain rate, 1/s	−0.32 ± 0.08	−0.14 ± 0.05^a^	−0.11 ± 0.06^a^	**<0.001**

*Note*: Data are reported as means ± standard deviation and were compared by ANOVA or chi‐squared test. Blood pressure and aortic distensibility are reported for 32 young adults. ^a^Significantly different from young control. ^b^Significantly different from old control. Abbreviations: BMI, body mass index; DBP, diastolic blood pressure; HFpEF, heart failure with preserved ejection fraction; HR, heart rate; MAP, mean arterial pressure; PP, pulse pressure; SBP, systolic blood pressure.

**FIGURE 2:**
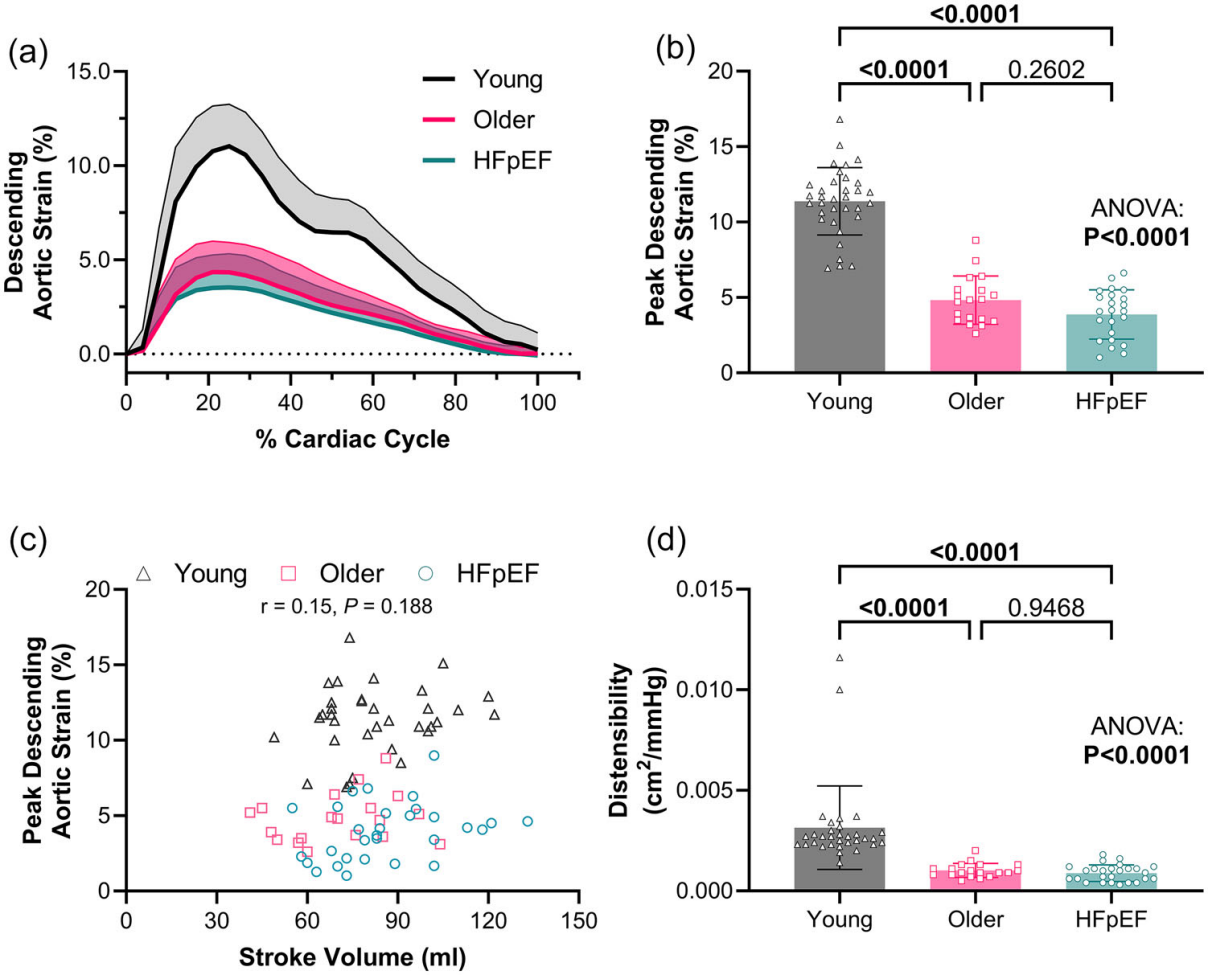
Descending aortic strain. (a) Average strain curves indexed to percentage of cardiac cycle duration, with the continuous thick line representing the mean and the shaded area representing the standard deviation. (b) Peak systolic strain was significantly different between young adults, older adults and patients with HFpEF. (c) There was no relationship between stroke volume and peak strain across all groups. (d) Aortic distensibility was significantly different between the young adults and both the older adults and patients with HFpEF. All data were compared via ANOVA with Tukey's *post hoc* test. HFpEF, heart failure with preserved ejection fraction.

Peak descending aortic strain was lower in the older and HFpEF groups when compared to the young (Table 1 and Figure 2b). We observed no significant correlation between stroke volume and peak strain (*r* = 0.15, *P *= 0.188; Figure 2c). Minimum and maximum aortic areas were greater in the older and HFpEF groups (both ANOVAs *P *< 0.001) with no difference between the older and HFpEF groups (Table 1). Both peak absolute descending aortic distension (maximum − minimum area; Table 1) and distensibility (Figure 2d) were lower in the older and HFpEF groups when compared to the young group (both ANOVAs, *P* < 0.001), with no difference between older and HFpEF groups. All between‐group differences remained when statistically adjusted for stroke volume, minimum and maximum aortic area, and mean arterial pressure (ANCOVA, *P *< 0.001).

**FIGURE 3:**
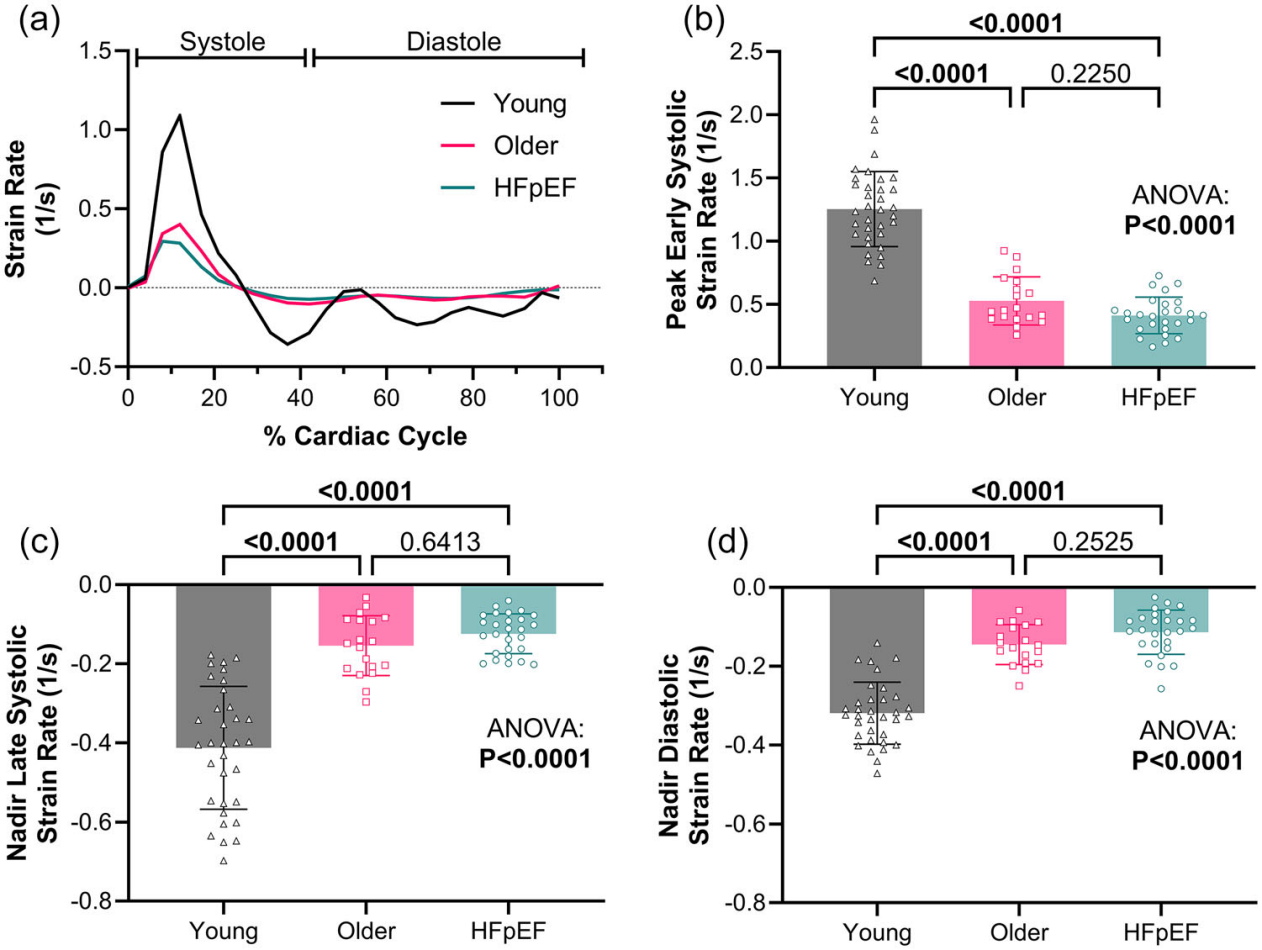
Descending aortic strain rates. (a) Average strain rate curves for each group in young adults, older adults, and adults with HFpEF. (b–d) Peak early systolic (b) and the nadir late systolic (c) and diastolic strain rates (d) were lower in young adults compared to both the older and HFpEF groups; there was no difference in strain rates between the older adults and adults with HFpEF. All data were compared via ANOVA with Tukey's *post hoc* test. HFpEF, heart failure with preserved ejection fraction.

Peak early and nadir late systolic strain rates were not different between the older and HFpEF groups, but both were greater in the young group (Table 1 and Figure 3b,c). Similarly, the nadir diastolic strain rate was greater in the young compared to the older and HFpEF groups, with no difference between the older and HFpEF groups (Figure 3d).

## 4 DISCUSSION

The primary finding of our study is that peak descending aortic circumferential strain and strain rates are sensitive to age. Peak descending aortic strain and strain rates may provide a new and valuable index of arterial stiffness for cross‐sectional and longitudinal studies utilizing CMR.

The assessment of arterial mechanics provides high‐sensitivity insight into arterial structure and function. We have previously observed habitual‐exercise‐related differences in carotid artery strain and strain rates (mechanics) that were not detected with conventional parameters of local arterial stiffness (Pugh et al., 2018). Herein, the between‐group differences with aortic distension (∆area), distensibility and strain were the same for all groups. Furthermore, both the older and HFpEF groups had lower strain and strain rates when compared to the younger adults studied here. However, we observed less variability for aortic strain compared to the conventional measures of area change and arterial distensibility. Notably, there were differences in aortic geometry (area) between the young and the older HFpEF groups which could contribute to differences in aortic strain and strain rate. However, the differences in peak systolic circumferential strain between groups remained following statistical adjustment for diastolic and systolic aortic area, as well as blood pressure and stroke volume suggesting minimal influence.

Here we utilize a similar approach to the one that has been performed in the assessment of cardiac function for many years, with the assessment of strain and mechanics. The technique has been applied to assessments of the carotid artery (Bjallmark et al., 2010; Black et al., 2016; Pugh et al., 2018; Talbot et al., 2020) and the descending aortic (Rong et al., 2020) stiffness using ultrasonography, but not with MRI. The tunica intima is often traced to assess the aortic area, which is plotted against time to interrogate the change in area across the cardiac cycle (Guala et al., 2019; Ohyama et al., 2018). However, the relative distension and associated rates of vessel distention and recoil (strain rates) are unexplored and represent the innovation of using the paradigm typically employed to interrogate cardiac function (Smiseth et al., 2024), which is considered to be less dependent on blood pressure. Whether vascular strain rates are also less influenced by arterial pressure remains to be investigated systematically. Importantly, we observe similar between‐group differences with peak aortic circumferential strain and both systolic and diastolic aortic strain rates in the current study.

Aortic strain and distensibility were not different between patients with HFpEF and older controls indicating that these groups present with similar age‐related arteriosclerosis when measured in the descending aorta, as was shown previously in the ascending aorta (Hundley et al., 2001) and carotid artery (Kitzman et al., 2013). Regional assessments of large elastic artery stiffness (carotid–femoral pulse wave velocity) have been reported to be higher in adults with HFpEF compared to healthy controls (Desai et al., 2009). Discordance between local and regional measures of arterial stiffness has been reported previously (Tanaka, 2018). The disparate findings dependent on technique may relate to whether blood pressure (pulse pressure or mean arterial pressure) is included in the calculation of the outcome variable. As such our findings of no difference in local descending aortic stiffness between patients with HFpEF and controls provide alternative and complimentary insight as to the effect of HFpEF on arterial stiffness in comparison to studies that measured regional carotid–femoral (aortic) pulse wave velocity.

### 4.1 Methodological considerations

A major benefit of the current four‐chamber approach to assessing descending aortic strain and mechanics is that no additional images are required beyond a standard CMR. Furthermore, this approach tracks the entire circumference of the arterial wall throughout the cardiac cycle, rather than assessing vascular distension via changes in diameter, which enables the calculation of overall and regional strain and systolic and diastolic strain rates, parameters that provide additional insight into vessel mechanics. However, the analysis presented here is retrospective in nature and as such does not allow for comparisons of descending aortic strain with other indices of arterial stiffness. Nevertheless, the aim of our analysis was to show the feasibility of the approach for future analysis of large datasets that have access to a range of assessments of arterial stiffness. While we included blood pressure, stroke volume and aortic area as covariates, other factors such as cardiac function, atherosclerosis and calcification could contribute to differences in circumferential strain and mechanics.

### 4.2 Conclusion

In conclusion, descending aortic circumferential strain and strain rates are easily obtainable outcomes from standard CMR that are sensitive to age. Future studies are required to measure descending aortic circumferential strain (and mechanics) in larger datasets to assess whether these novel parameters are associated with clinical outcomes in health and disease.

## AUTHOR CONTRIBUTIONS

Conception or design of the work: Denis J. Wakeham, Sauyeh K. Zamani, Christopher M. Hearon, Satyam Sarma, Michael D. Nelson Acquisition, analysis or interpretation of data for the work: Denis J. Wakeham, Sauyeh K. Zamani, Andrew Oneglia, Matthew M. Howrey, Samer Majeed, Tiffany L. Brazile, Joshua A. Beckman, James P. Macnamara, Mark J. Haykowsky, Vlad G. Zaha, Benjamin D. Levine, Christopher M. Hearon, Satyam Sarma, Michael D. Nelson Drafting the work or revising it critically for important intellectual content: Denis J. Wakeham, Sauyeh K. Zamani, Andrew Oneglia, Matthew M. Howrey, Samer Majeed, Tiffany L. Brazile, Joshua A. Beckman, James P. Macnamara, Mark J. Haykowsky, Vlad G. Zaha, Benjamin D. Levine, Christopher M. Hearon, Satyam Sarma, Michael D. Nelson. All authors have read and approved the final version of this manuscript and agree to be accountable for all aspects of the work in ensuring that questions related to the accuracy or integrity of any part of the work are appropriately investigated and resolved. All persons designated as authors qualify for authorship, and all those who qualify for authorship are listed.

## CONFLICT OF INTEREST

None declared.

## Data Availability

The data that support the findings of this study are available from the corresponding author upon reasonable request.
